# [Retracted] Endothelial cell-derived exosomes protect SH-SY5Y nerve cells against ischemia/reperfusion injury

**DOI:** 10.3892/ijmm.2025.5549

**Published:** 2025-05-12

**Authors:** Bing Xiao, Yi Chai, Shigang Lv, Minhua Ye, Miaojing Wu, Liyuan Xie, Yanghua Fan, Xingen Zhu, Ziyun Gao

Int J Mol Med 40: 1201-1209, 2017; DOI: 10.3892/ijmm.2017.3106

Following the publication of this paper, it was drawn to the Editor's attention by a concerned reader that the flow cytometric data shown in Figs. 3A and 5E, and certain of the EdU assay data shown in Fig. 5C were strikingly similar to data that had either already appeared in previously published articles that were written by different authors at different research institutes, or were featured in articles that were submitted for publication to different journals at around the same time, or have otherwise subsequently appeared in print elsewhere. Owing to the fact that the contentious data in the above article had already been published prior to its submission to *International Journal of Molecular Medicine*, the Editor has decided that this paper should be retracted from the Journal. The authors were asked for an explanation to account for these concerns, but the Editorial Office did not receive a reply. The Editor apologizes to the readership for any inconvenience caused.

